# RS–DFID Africa capacity-building initiative programme grant: harnessing unsteady phase-change heat exchange in high-performance concentrated solar power systems

**DOI:** 10.1098/rsfs.2023.0059

**Published:** 2024-08-09

**Authors:** Benedict Winchester, Abdullah M. Maghrabi, Christos N. Markides

**Affiliations:** ^1^ Clean Energy Processes (CEP) Laboratory and the Sargent Centre for Process Systems Engineering, Department of Chemical Engineering, Imperial College London, London SW7 2AZ, UK; ^2^ Department of Physics, Imperial College London, London SW7 2AZ, UK; ^3^ Grantham Institute – Climate Change and the Environment, Imperial College London, London SW7 2AZ, UK

**Keywords:** capacity building, concentrated solar power, direct-steam generation, renewable energy, solar power

## Abstract

The Royal Society and UK Department for International Development supported a consortium of three universities across sub-Saharan Africa and Imperial College London with the aim of developing new knowledge on direct-steam-generation concentrated solar power (CSP) plants and supporting relevant capacity building across the Universities of Lagos, Mauritius and Pretoria. Key research findings from the programme include an improved flow-classification scheme for two-phase, liquid–liquid flows; testing of advanced surfaces with much-improved steady-state heat transfer performance—the commercial nanoFLUX surface showed up to 200% higher heat-transfer coefficients (HTCs) in pool boiling compared with other surfaces with R-134a/R-245fa; first-of-a-kind measurements of transient flow boiling HTCs, which were up to 30% lower in step perturbations than quasi-steady-state expectations in horizontal pipes with R-245fa; error estimation and corrections for laser-induced fluorescence (LIF) measurements, leading to the development of an adapted planar LIF technique with uncertainty <10% for local, instantaneous film thickness measurements in annular flows, and the application of such diagnostic methods to pool, falling-film and flow boiling in pipes; and predictions of an ~80% increase in the net present value of a case-study CSP plant when integrated with solid storage media.

## 1. Introduction and project motivation

**Figure 1.**
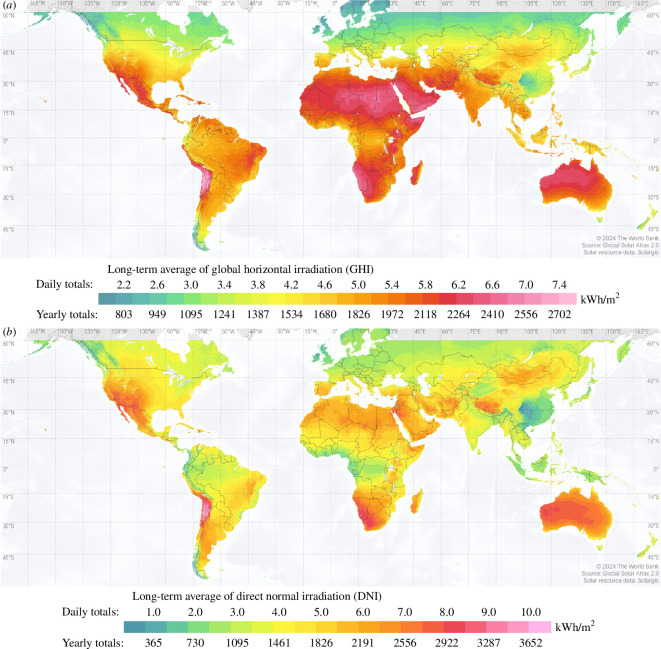
World maps of (*a*) global horizontal irradiation (GHI) and (*b*) DNI. Africa, in the centre of the map, has a high solar resource, visible in the dark-red shading on the map. (Source: Marcel *et al*. [3].)

Concentrated solar power (CSP) for electricity generation is a promising renewable-energy technology, especially in global regions with rich solar resources [1,2]. Figure 1 shows world maps of global horizontal and direct normal irradiance (DNI) reproduced from Marcel *et al*. [3]. From these maps and the literature, it is clear that Africa, especially sub-Saharan Africa (SSA), has an excellent potential for the deployment of solar technologies in general and CSP in particular [4–7]. Such a possibility would have multiple positive effects on the region, supporting and accelerating sustainable economic development through access to reliable electricity, while improving the lives of people, and ensuring that environmental sustainability is not jeopardized, in line with the United Nations’ Sustainable Development Goals (SDGs), SDG 7 in particular [8].

**Figure 2.**
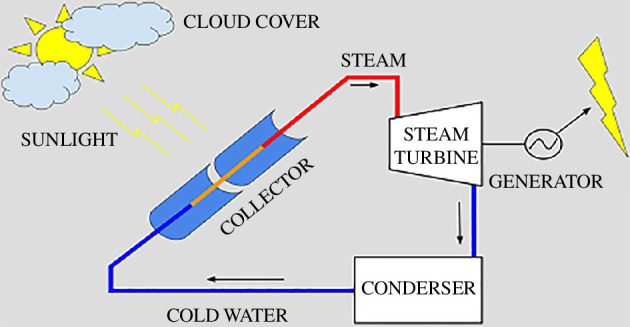
Schematic of a DSG-CSP plant. Water is used as the heat-transfer fluid in the solar collectors, and is heated to generate steam directly within the pipes in the solar field. This steam is then used to drive a turbine to generate electricity in the power block [10].

An interesting and much-promising CSP option that has gained growing interest in recent decades is based on direct steam generation (DSG) [9]. DSG-CSP plants have some advantages over conventional CSP plants, which have been developed and deployed for the generation of power from sunlight as shown in figure 2. While other plants use secondary heat-transfer fluids (HTFs) in the solar fields (e.g. thermal oils or molten salts and depending on the temperature) and heat exchangers to generate steam for power generation, DSG plants produce steam directly in the solar field as the HTF used here is the same as the working fluid that is used in the thermodynamic power cycle that produces electricity (i.e. water–steam) [9]. Some advantages of DSG plants over indirect steam-generation CSP plants that arise from this design approach include higher steam temperatures [11,12], improved overall plant efficiencies [13], simplified designs, which are easier to maintain, though potentially more complex [14], reduced pumping-power requirements and reduced loss of HTF (through leaks) to the environment.

At the same time, such SDG-CSP plants operate under strongly varying conditions with a reduced ability to control this time-varying operation [15,16]. Owing to variations in cloud coverage, ambient conditions and diurnal irradiance, there will be variations in the flow as well as unsteadiness in the heat transfer (and steam generation rate) in the solar field [17,18]. While research has been dedicated to understanding DSG collectors under steady-state operation [19,20], the operation of these plants under unsteady conditions remains relatively unexplored, with a lack of knowledge on performance during transient operation.

To contribute to the knowledge and advancement of DSG-CSP technology, and to influence local development in Africa, a region with high potential for CSP [7–9,11], by improving clean, renewable electricity access and stimulating economic growth, a 5-year research programme titled ‘Harnessing unsteady phase-change heat exchange in high-performance CSP systems’ was supported by the United Kingdom Department for International Development (DFID) through the Royal Society [10]. This programme grant was one of 10 programmes supported, in two tranches of five projects each, as part of the £15 million Africa Capacity Building Initiative (ACBI), which focused on activities across three main areas: water and sanitation; renewable energy; and soil-related science. The primary objectives of the DSG-CSP project were to conduct fundamental research using advanced experimental and numerical methods on flow with phase change (boiling and condensation), heat transfer in the presence of unsteadiness, and to use new data collected from fluorescence experiments to develop models that can describe transient plant performance. In this article, we present a summary of this project, highlighting both its scientific and capacity-building and -strengthening achievements.

## 2. Project aims, objectives and structure

The ‘Harnessing unsteady phase-change heat exchange in high-performance CSP systems’ project funded by the Royal Society–DFID ACBI programme was proposed by a consortium consisting of researchers from Imperial College London (the United Kingdom), the University of Lagos (Nigeria), the University of Mauritius (Mauritius) and the University of Pretoria (South Africa), with a total project budget of approximately one million British pounds.

The following high-level project aims were identified:

— *Novel research*: establishing partnerships and undertaking the necessary underpinning multidisciplinary research with academic institutions in SSA.
— *Capacity building*: establishing research, human and physical (i.e. laboratory and computational) support and training capacity in the region, along with the infrastructure and knowledge to support future research and development.

Each consortium member institution consisted of a principal investigator (PI), a PhD student, as well as other co-investigators, technicians, researchers and/or students as required. Specifically, the team at the University of Lagos was led by Dr Olabode Olakoyejo, who was supported by academics Drs Adekunle Adelaja and Olanrewaju Obayopo, while the team at the University of Mauritius was led by Dr Mohammad Elahee, supported by Dr Muhammad Dauhoo and Mr Abdel Khoodaruth. Finally, the PI at the University of Pretoria was Professor Josua Meyer, who was supported by Dr Jaco Dirker who, like the other co-investigators, provided additional guidance, mentorship and co‐supervision to the students.

**Figure 3.**
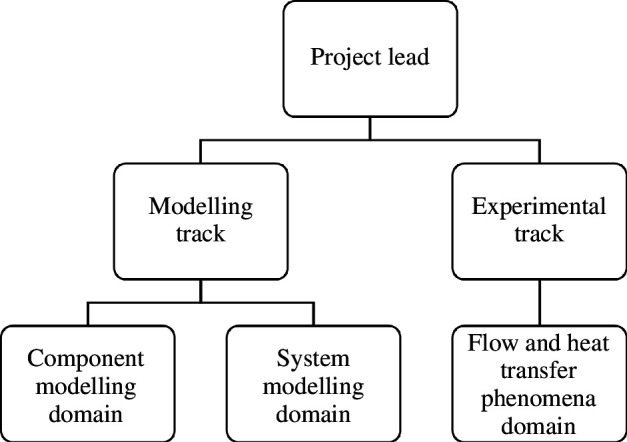
Schematic of the project structure. The project is divided into two main themes: modelling and experiments. Modelling was subdivided into component- and system-level modelling activities, while the experimental branch of the project investigated the flow- and heat-transfer phenomena.

The project structure of the research is shown in figure 3. The structure comprised two main tracks that were led by the overall lead of the project, Professor Christos Markides, based at Imperial College London:

— *Modelling*: within which component- and power-system-level models were developed to better represent the performance and operation characteristics with a focus on the (less understood) transient behaviour of DSG plants; improved understanding of the stability and operation of DSG plants through modelling to improve the overall technological feasibility.
— *Experiments*: which focused on understanding the fluid-flow and thermal-energy processes that take place during flow boiling, condensation and thermal-energy storage that are key elements of DSG-CSP plants. These processes are highly complex, multiscale and multi-physics in nature, and involve phase-change, unsteady and turbulent multiphase flows in the presence of conjugate heat transfer [21].

The capacity-building aspects of the project are covered in §3, while the novel research outputs of the grant are summarized in §4.

## 3. Capacity-building approaches and achievements

The capacity-building aspects involving the African partner institutions remained an important component of the project throughout the funding period. Overall, this element was approached from both human-capital training and development and assets-development aspects, covering research capacity at the partner institutions and extending to support and capacity in the wider region.

With respect to the human-capital capacity building, beyond the investigators, technical and support staff at the African universities, the project involved the training of three doctoral students, one from each of the African universities involved. Training took place both in the UK and in the African universities. Initial skills transfer was performed mainly via interactions with Imperial College London using core team members from each African university, which visited the UK annually. Topic experts, e.g. on software platforms or measurement equipment, were brought in during a series of workshops held at the various universities. Formal training included the following elements: (i) technical experimental-related training; (ii) technical numerical-related training; and (iii) vocational training. Furthermore, the consortium held a number of annual meetings, interacted with policy and energy sector experts and also included a visit and tour of CSP plants (a 50 MW solar power tower and a 100 MW parabolic trough solar thermal power plant) near Upington in South Africa.

**Figure 4.**
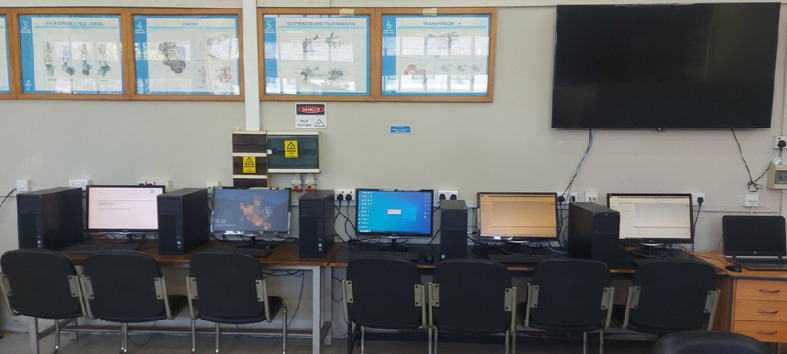
Numerical modelling and simulation cluster at the University of Mauritius.

This resulted in local CSP expertise being developed at each of the institutions, training individuals who are well equipped with up-to-date technical skills and broader academic and scientific skills. Moreover, the project fostered the establishment and development of relations and channels for interactions between participating universities for further international collaboration. In addition to the human-capital capacity building, several research-related assets were established as part of this project, of which two are computational laboratories, one in Lagos and one in Mauritius (shown in figure 4), with the ability to perform complex simulations of multiphase flows with heat transfer.

At the University of Mauritius, numerical models were developed for the simulation of two-phase condensing flows. These models were used to generate numerical data of such flows of interest, with a focus on CSP applications, over a range of relevant conditions. Additionally, a high-performance simulation cluster was established. At the University of Lagos, the focus was on developing numerical models of two-phase boiling/evaporative flows in pipes, and similar research to that in Mauritius was undertaken but for flow boiling. At Lagos, the establishment of the computational laboratory included backup generators to avoid time and data losses due to intermittent power availability.

**Figure 5.**
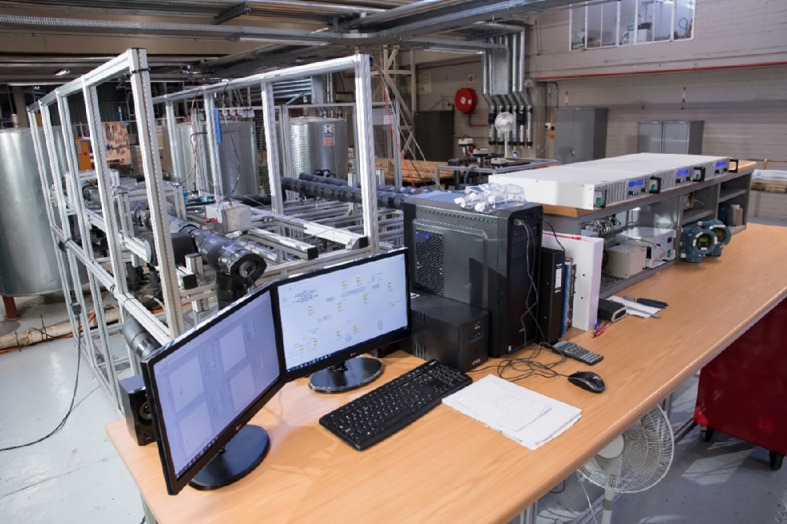
Flow boiling facility at the University of Pretoria.

At the University of Pretoria, existing experimental facilities for the study of flow condensation (shown in figure 5) were expanded under the grant to enable the generation of flow boiling data, which allowed the generation of extensive experimental data of these flows under transient conditions. The data were used both in their own right, for new knowledge and to develop new designs, and also to support the development and validation of models and numerical codes. Finally, at Imperial College London, existing experimental facilities were extended to allow for the study of flow-boiling heat transfer. This included expansion of the laser-based diagnostics capabilities as applied to these flows, which can provide a unique insight into the underlying (fluid-mechanical and thermal) space- and time-resolved processes. Tangible benefits to Africa include the promotion of energy resilience and independence, expanded knowledge and skills, improved economic conditions and competitiveness, both directly from the research outputs, but also via the training and development of personnel, infrastructure and resources.

## 4. Key research findings and observations

The research component of the project was a core element of the programme. Here, we summarize some key research outputs of the project and the impacts achieved.

### 4.1. Challenges and gaps in two-phase phase-change flows

Natural and industrial processes ranging from small-scale ones used, for example, in the medical and pharmaceutical industries and food production, to the large-scale transport of crude oil in pipelines and those that appear in CSP plants owing to phase change (i.e. boiling and condensation, in different parts of such plants), include the simultaneous appearance of two (or more) fluid phases in the same conduit. Owing to the wide range of different parameters and conditions that are achievable in such non-isothermal two-phase flows, various flow patterns can be obtained, with the resulting flow regime being critical in controlling the overall flow and heat transfer characteristics.

A review of the various flow conditions and phenomena that are involved in DSG solar fields for CSP power was undertaken by Dirker *et al.* [21]. This review identified several gaps in the existing literature that need attention in order to build a coherent and holistic picture of the underlying physics involved in DSG-CSP plants and to develop a set of reliable, accurate tools for the design of next-generation systems with improved operation and performance:

— Flow regimes and their *a priori* identification for design purposes, discussed also below (§4.2) in terms of a new classification scheme developed, are difficult to predict, yet their impact on the thermal performance of key components is critical. Flow-regime/pattern maps and statistical (probabilistic) methods are common approaches that have been used traditionally for the prediction of these regimes; however, their modelling and classification remain a research gap, especially in the context of transients that were of interest in the project.
— Challenges in flow boiling and condensation:
  - Reliable predictions of the thermal performance—quantified by the heat-transfer coefficient (HTC)—are challenging at the scales involved in DSG-CSP systems, even though advancements are being constantly made to a range of models;
  - The pressure drops in channels where these flows occur are also an aspect where uncertainty exists, although, in the context of CSP applications, this is less critical than heat transfer. Nevertheless, modelling and experiments can be used to improve our understanding and predictive abilities, especially at the higher mass flow rates required in real applications;
  - Considerable uncertainty remains in predictions of both heat transfer and pressure drops in relevant components under the influence of system transients, e.g. irradiance, ambient temperature, flow rate, etc.
— At the higher radiative heat fluxes found in CSP systems, there are uncertainties in the fluid properties and also in the dynamics of the multiphase flows within the collector field and subsequent piping.

### 4.2. New insights via high-fidelity flow measurements

Liquid–liquid flows are a class of two-phase flows that were of interest early on to the consortium as they present the experimentalist with fewer challenges in the application of optical measurement techniques, and, thus, were used as a natural first step in the development of a range of optical measurement methods of interest before these were applied to more complex two-phase flows. In Ibarra *et al.* [22], a comprehensive effort was made to review and synthesize available information on liquid–liquid flows in the existing literature, and to develop a unified classification scheme for these flows. This required the identification of key parameters (including pipe characteristics, flow velocity and fluid properties) based on which a new classification system was proposed featuring the following:

— Stratified flows, where the two immiscible liquids flow over each other with the less dense liquid flowing above the denser liquid.
— Stratified wavy flows with droplets at the interface, where an increase in the flow results in the formation of small droplets in the flow at the interface between the two immiscible liquids.
— Dispersion of liquid phases, from one phase to the other:
  - Dispersion of the less dense liquid (e.g. oil) in the denser liquid (e.g. water), where the denser liquid flows continuously and the less dense liquid flows purely as droplets suspended in the denser flow, which can be with:
    - a continuous, pure, denser (water) layer;
    - or with no pure water layer, i.e. droplets dispersed throughout.
  - Dispersion of the denser liquid (e.g. water) in the less dense liquid (e.g. oil), where the less dense liquid flows continuously and the denser liquid flows as suspended droplets:
    - again, this can be with a continuous, pure, less dense (oil) layer;
    - or this can be with no pure oil layer, i.e. droplets of water are dispersed throughout the oil.
  - Dual-continuous flow, where both liquids flow both continuously and as a suspension of droplets in the opposite phase.
— Intermittent flows, where ‘slugs’ disperse such that the flow consists of a continuous flow of one of the liquids with the other interspersed in large droplets.
— Annular flow, where one liquid flows at the centre of the pipe and the other flows around the edge, forming a pattern with a ‘core’ liquid and an ‘annular’ liquid.

Of importance is the fact most of these flow patterns and regimes also appear in non-isothermal two-phase flows in the presence of phase changes, which is strictly the type of flow that is relevant to CSP systems (both boiling and condensation).

The classification system focuses specifically on non-dimensional parameters allowing for a more straightforward ‘scale-up’, or transfer of knowledge across scales. This is important when attempting to extrapolate and make the results of laboratory-scale measurements relevant to large engineering systems such as CSP plants.

Delving further into the application of detailed optical techniques, and expanding out to consider solids, researchers at Imperial investigated refractive index matching (RIM) in the context of optical measurement methods [23]. RIM is a technique of particular relevance to multiphase flows, which may contain fluids of different refractive indices, thus giving rise to optical distortions that affect the optical imaging of these flows. Optical methods can be used to characterize the properties of the various flows and to probe various flow characteristics. There are several optical measurement techniques for which RIM is relevant:

— Laser-induced fluorescence (LIF), where a laser is used to excite a fluid and cameras capture the light emitted (fluoresced) from the fluid.
— Laser Doppler velocimetry (LDV), using the Doppler shift on reflected light from a laser when it encounters particles suspended in the fluid to examine fluid velocity.
— Particle image velocimetry (PIV), where image sequences are used to track the movement of particles, which are suspended in a fluid to determine the position and, therefore, velocity of the flow.
— Particle tracking velocimetry (PTV), which is similar to PIV, but with individual particles identified and tracked rather than particle groups.

These optical measurement methods are non-intrusive and can provide high-accuracy, whole-field space- and time-resolved information which is otherwise difficult to obtain. The project was motivated by the capability of these methods to provide new, previously unavailable information in the flows of interest, expanding our insight into the underlying thermal-fluid processes, but also providing a unique database of the development and validation of advanced computational prediction codes and design tools. Wright *et al*. [23] considered solutions, e.g. combinations of solid and liquid phases, that can allow the minimization of optical distortions and resulting errors when attempting to apply these optical measurements in a range of multiphase flows. The consortium was successful in applying—for the first time—LIF-based and PIV/PTV methods to boiling flows in horizontal pipes [24,25]. We will return to these results later in §4.3, where we outline the key findings and contributions made to the field by the consortium during the remit of this project.

### 4.3. Novel findings and contributions

The consortium focused its efforts mainly onto boiling heat-transfer phenomena in pipes, as this is of direct relevance to DSG-CSP. Nevertheless, the scaled-down nature of the investigated flows and alternative fluids used enable us to transfer knowledge to other flows and to benefit other applications across scales, e.g. to phenomena relevant to low-grade heat conversion but also refrigeration applications. In this section, we summarize the consortium’s findings arising from both experimental (§§4.3.1–4.3.5) and computational (§4.4) research approaches.

#### 4.3.1. Isothermal two-phase gas–liquid flows

Having developed a selection of optical measurement techniques and applying these first to liquid–liquid flows, LIF was then applied to a range of gas–liquid flows. This was done again in gradual steps of flow and optical complexity, starting from isothermal gas–liquid (i.e. air–water) flows (covered in this section), to pool boiling (in §4.3.2), falling-film boiling (in §4.3.3) and finally flow boiling in pipes (in §§4.3.4 and 4.3.5).

**Figure 6.**
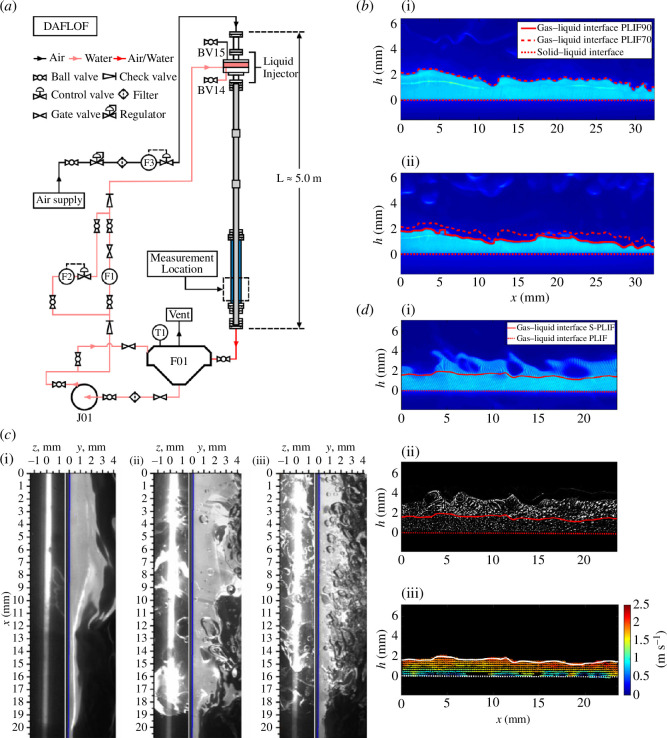
(*a*) DAF apparatus and flow-monitoring equipment, reproduced with permission from Cherdantsev *et al.* [26]; (*b*) S-PLIF images of waves in flows with gas and liquid Reynolds numbers of (i) 0 and 1505, (ii) 22 100 and 1363 and (iii) 48 300 and 1636, reproduced with permissiAbstracton from Charogiannis *et al.* [27]; (*c*) raw, unprocessed BBLIF (left) images and PLIF (right) of waves in flows obtained with gas and liquid Reynolds numbers of (i) 34 900 and 300, (ii) 43 700 and 300 and (iii) 52 400 and 300, respectively, reproduced with permission from Cherdantsev *et al.* [26]; and (*d*) simultaneous measurements: (i) S-PLIF, (ii) PIV, and (iii) two-dimensional velocity map in a flow with liquid and gas Reynolds numbers of 1200 and 0 [27].

In more detail, the consortium first turned its attention to isothermal annular air–water flows, with the main findings reported in [26,27]. A selection of key results from these papers is shown in figure 6. In [26], two LIF techniques, namely planar LIF (PLIF) and brightness-based LIF (BBLIF), were applied simultaneously to a range of downward annular flows (DAFs). The schematic in figure 6*a* shows the flow facility and DAF apparatus with the inlet section, main pipe, RIM correction box and the wider loop containing the tank, pump and mass flow controllers. Experiments were performed over liquid Reynolds numbers in the range 140−1330 and gas Reynolds numbers in the range 0–85 100. From the generated data, it was found that total internal reflection (TIR) when using PLIF can lead to an overestimation of the film thickness in annular flows, while using BBLIF for these measurements can be affected by local losses of signal sensitivity in thick film regions and the presence of steep phenomena (such as wave fronts) or multiple interfaces (such as in agitated flow regions) leading to errors in the identification of the liquid–gas interface location. The combined information from the two methods allowed the authors to reduce errors associated with the separate application of individual methods, and to focus on the air–water interface and its dynamics with greater accuracy.

Motivated by the errors identified earlier, a new technique was developed that we refer to as ‘structured planar laser-induced fluorescence’ (S-PLIF), and this was reported in [27]. S-PLIF relies on the spatial, periodic variation in the intensity of the laser sheet used, and was validated and explored in the context of film thickness measurements in similar annular flows to those explored in [26].

#### 4.3.2. Pool boiling

**Figure 7.**
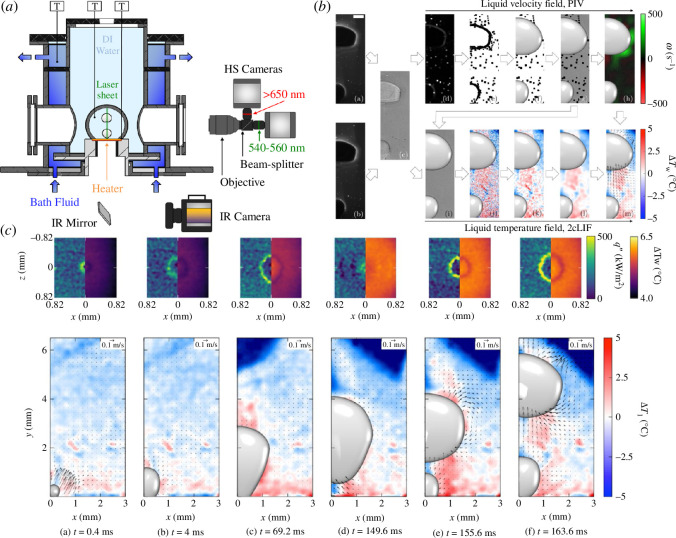
(*a*) Apparatus and optical measurement arrangement, (*b*) image analysis process and (*c*) simultaneous results with the top row showing heat flux (left) and temperature (right) maps obtained through IR diagnostics, and the bottom row showing temperature and velocity fields in the liquid phase obtained with 2cLIF and PIV, respectively. Taken from Voulgaropoulos *et al.* [28].

Of particular interest in boiling are the bubble dynamics, from the bubble nucleation process at a nucleation site, bubble growth, and detachment into the liquid bulk, which are crucial in controlling the overall heat transfer rate. Combining PLIF (a two-colour technique was used in this instance) with PIV and infrared (IR) thermometry, Voulgaropoulos *et al.* [28] were able to study the process of bubble dynamics in the pool boiling of water. Results from this study are shown in figure 7. The study measured bubble growth rates and detachment sizes, liquid microlayers and reported with these techniques for the first time that, when a bubble detaches, liquid mixing occurs behind it, thus promoting ‘rewetting’, a process of liquid covering the nucleation site.

The apparatus shown in figure 7, used in the experiment, consists of a boiling cell (operating at atmospheric pressure), which is filled with a solution of deionized water and the fluorophores—fluorescent compounds—used for the laser-induced techniques. A warm fluid was circulated around the cell to bring it to saturation temperature. Optical access was via four quartz windows, which enabled the high-speed cameras (pictured on the right of figure 7*a*) and the laser-induced techniques (pictured on the bottom of figure 7*a* to access the chamber. The temperature was monitored also through two thermocouples.

**Figure 8.**
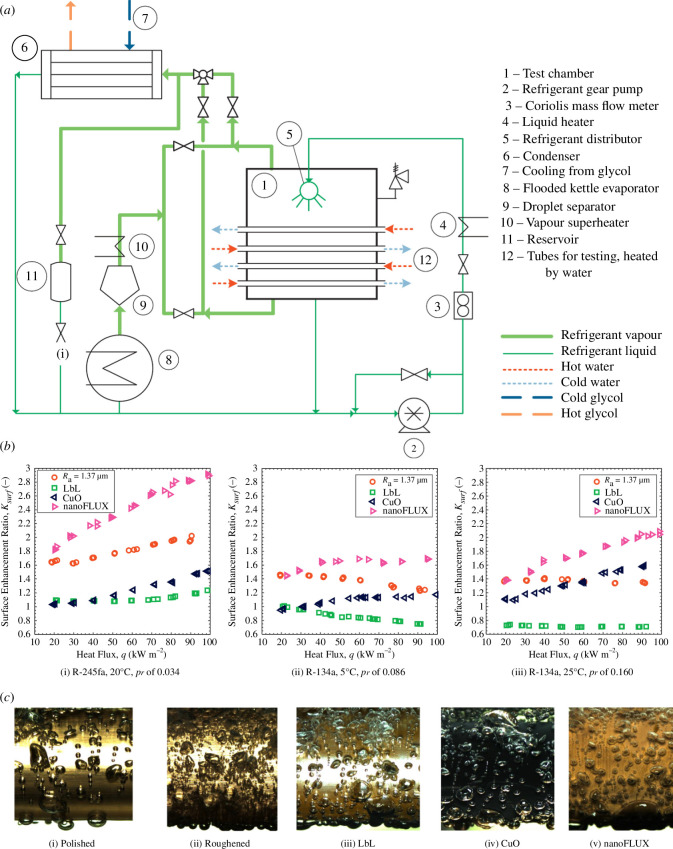
(*a*) Apparatus, (*b*) measured HTCs as a function of heat flux for the three investigated pipe surfaces (i.e. polished, roughened and nanostructured tubes) with the flow conditions shown, and (*c*) images of R-245fa boiling over the surfaces at 20 kW m^−2^ and 20°C. Taken from Bock *et al*. [29].

Further efforts by the consortium focused on the pool boiling of refrigerants relevant to a wider range of applications, specifically R-134a (at 5 and 25°C) and R-245fa (at 20°C), and over a range of heat fluxes from 20 to 100 kW m^−2^ [29]. Specifically, Bock *et al.* [29] investigated pool boiling over tubes with three surfaces: applying nanocoatings to polished copper using a layer-by-layer approach, a chemical oxidation process, which resulted in sharp copper oxide structures on the surface, and a commercial process known as nanoFLUX. Results from the study, including the equipment used, are shown in figure 8. The apparatus consists of tubes in a test chamber, submerged in a pool of liquid refrigerant, with vapour travelling to the overhead condenser during boiling. The resulting condensate is returned to the liquid pool in the test chamber via a refrigerant pump. The saturation pressure of the refrigerant was measured using pressure transducers and thermocouple readings to confirm that a minimal quantity of non-condensable gases was present.

The authors found that the commercial nanoFLUX process had up to 200% higher HTCs compared with the other approaches, but that the copper-oxide and nanoFLUX approaches showed the highest variability in the HTC compared with the nanocoating approach. All three surfaces showed power-law relationships with similar HTCs at reduced pressure values of 0.034, which differed as the reduced pressure was increased. At the highest reduced-pressure values studied (0.086 and 0.160), the oxidized surface outperformed the rough (sharp) surface though the nanoFLUX surface had the highest HTCs at all values of the reduced pressure.

#### 4.3.3. Film boiling

Over the last decade, the team at Imperial has undertaken extensive research on the application of optical diagnostic techniques (specifically, combined LIF, PIV and IR measurements) to heated falling films. However, these were restricted to lower heat fluxes (i.e. evaporation) rather than boiling [30–32]. The AFCI project was a unique opportunity to consider boiling at higher heat fluxes.

**Figure 9.**
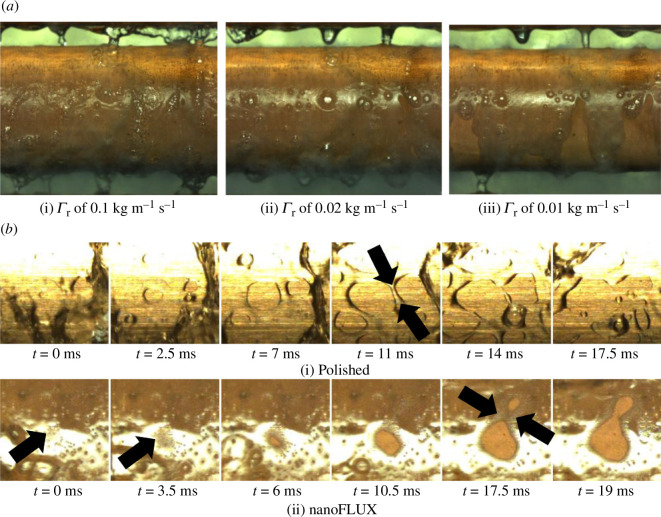
(*a*) Heat-transfer hump for R-134a boiling over the nanoFLUX tube at 20 kW m^−2^, 25°C and three different mass fluxes: (i) 0.1, (ii) 0.02 and (iii) 0.01 kg m^–1^ s^–1^; (*b*) dry-spot progression for both polished (top row) and nanoFLUX (bottom row) surfaces. Taken from Bock *et al*. [33,34].

Building on the categorization work of the heat-transfer effects of pool boiling over a range of nanostructured tubes [29], the same authors extended their investigations into falling-film boiling. The authors used tubes, placed horizontally within a test chamber, with a thin film of refrigerant distributed along the length of the outside of the tubes. This film was heated by water, which flowed within the tubes. The tubes had an external diameter of 19.05 and a spcaing of 3.25 mm between them. The apparatus is outlined in more detail in Bock *et al.* [33] and images captured during the study are shown in figure 9.

Bock *et al.* [33] considered falling-film evaporators, which use surfaces constructed using three approaches: a nanostructured tube; copper-oxide through chemical oxidation and the commercial nanoFLUX approach—also discussed in §4.3.2 and outlined in Bock *et al.* [29]. Here, however, the authors investigated the effect of varying the mass flow rate from 0 to 0.13 kg m^−1^ s^−1^, Reynolds numbers from 0 to around 1500 to 2500, and heat fluxes from 20 to 100 kW m^−2^. The authors concluded that the copper-oxide and nanoFLUX tubes reached critical dryout at the lowest Reynolds number. In some cases, as the heat flux was increased, a departure from nucleate boiling became the limiting factor. In general, falling-film enhancement ratios were reported as typically reaching values of 0.8−1.3 in this work.

#### 4.3.4. Unsteady flow boiling in horizontal pipes

**Figure 10.**
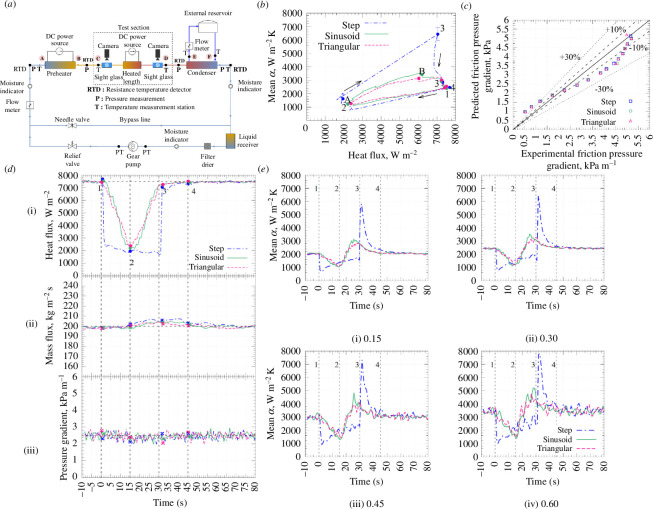
(*a*) Apparatus, (*b*) HTC as a function of the heat flux for imposed step (grey dash-dotted line), sinusoidal (green solid line) and triangular (red dashed line) heat-flux waveforms, (*c*) comparison of measured pressure drop against predictions by the Müller–Steinhagen and Heck [36] correlation, (*d*) results following step, sinusoidal and triangular heat-flux perturbations in a flow with an initial vapour quality of 0.30, mass flux of 200 kg m^−2^ s^−1^ and heat flux of 7.5 kW m^−2^: (i) heat flux, (ii) mass flux and (iii) pressure gradient, and (*e*) HTC response to step, sinusoidal and triangular heat-flux perturbations in flows with four different vapour qualities. Taken from Van den Bergh *et al.* [35].

Proceeding to experiments on flow boiling, which would be the closest to a DSG (or, indeed the other power generation or refrigeration applications mentioned earlier), the heat transfer and pressure drop of boiling flows in horizontal pipes were studied experimentally by Van den Bergh *et al.* [35]. The external conditions are known to play a controlling role in boiling heat-transfer performance, with a change in the boiling process having significant impacts on the heat transfer and evaporation rate; however, data on flow boiling in pipes under variable external conditions are lacking. A selection of noteworthy results from this article is shown in figure 10.

CSP plants enable electricity generation and district-scale heating, whether space heating or hot-water provision, for communities and industrial processes. The concentrated sunlight enables high temperatures to be reached; however, intermittent cloud coverage can significantly impact the performance of these plants, especially if these are based on DSG technology (where boiling takes place directly in the solar field). Intermittent cloud coverage results in a drop in heat flux applied to the working fluid. As an analogue to intermittent cloud coverage, Van den Bergh *et al.* [35] investigated the impact of different temporal heating patterns with electrical heating applied onto a horizontal pipe. They examined step-, triangular- and sinusoidal-waveform heating patterns and studied the effects that these heating variations had on the heat transfer and the pressure drop in the pipe.

The apparatus shown in figure 10 consists of a closed refrigerant loop containing R-245fa. The subcooled liquid was pumped and circulated through the electrically heated pre-heater (shown in the top-left of figure 10*a*) before passing through the heated test section and a water-cooled condenser. The inlet vapour quality was controlled using the test section wherein diabatic thermal measurements were performed. A condenser was used to remove heat that was added to the fluid in the preheater and test sections so that the fluid returned to a subcooled liquid state. Apart from the test section, all of the piping was smooth copper with an outer diameter of 12.7 mm.

Two results are of particular interest: the authors observed that a step function for the heating profile reduced the HTC by approximately 30% compared with the expectations from steady-state heating, with no impact on the pressure drop through the pipe section being examined. For triangular and sinusoidal heating patterns, they found that the HTC was reduced by 8%, again with no impact on the pressure gradient.

**Figure 11.**
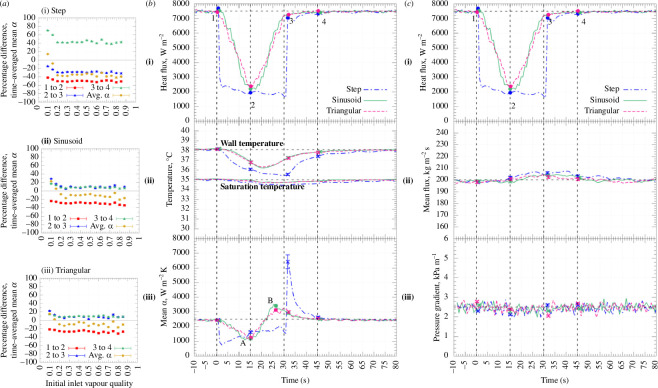
(*a*) Maximum average difference in the mean HTC for the different stages of the perturbation—‘1’ corresponds to pre-perturbation results, ‘2’ to a time midway through the perturbation, ‘3’ to the end of the perturbation and ‘4’ is 15 s after the perturbation—compared with the steady-state value for a mass flux of 200 kg m^−2^ s^−1^, and initial heat flux of 7.5 kW m^−2^: (i) step, (ii) sinusoidal and (iii) ramp perturbations, (*b*) step, sinusoidal and triangular heat-flux perturbations along with the response of variables measured for a vapour quality of 0.30, a mass flux of 200 kg m^−2^ s^−1^, and initial heat flux of 7.5 kW m^−2^: (i) applied heat flux, (ii) wall and saturation temperatures and (iii) mean HTC, and (*c*) step, sinusoidal and triangular heat-flux perturbations along with the response of variables measured for a vapour quality of 0.30, mass flux of 200 kg m^−2^ s^−1^, and initial heat flux of 7.5 kW m^−2^: (i) applied heat flux, (ii) mass flux and (iii) pressure gradient. Taken from Van den Bergh *et al*. [35].

Beyond cloud coverage, other parameters, such as the flow rate, can have a significant impact on flow boiling in DSG plants. Van den Bergh *et al.* [35] investigated the impact of the flow rate and heating intensity for R-245fa, a popular refrigerant proposed as a working fluid for organic Rankine cycle (ORC) systems. Key findings from this work are shown in figure 11. While there exists a wide body of existing research in this field, relatively low heat fluxes (<5 kW m^−2^) and low mass fluxes (<60 kg m^−2^ s^−1^) have not been thoroughly studied and are hence not well represented in the literature.

It was found that both the heat and the mass fluxes of the fluid had a significant impact on the HTC in the investigated system. Specifically, an increase in the mass flux resulted in an increase in the HTC, as well as an unexpected effect: at low heat fluxes, an extreme sensitivity to the mass flux was identified when the vapour quality was between 20% and 30%. A more detailed understanding of the thermal performance at these mass- and heat-flux regimes broadens our understanding of this field. While the results are novel, as these regimes are relatively newly explored, further studies are needed to investigate the phenomena at these low-flux regimes.

#### 4.3.5. Flow regimes and detailed measurements of flow boiling in pipes

**Figure 12.**
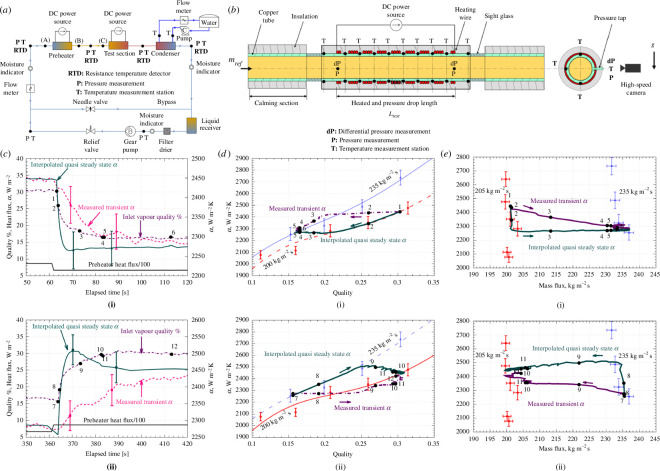
(*a*) Apparatus, (*b*) heated test section detail and (*c*) temporal evolution of the vapour quality, and heat flux and HTC owing to (i) downward and (ii) upward step changes in inlet vapour quality, for an initial vapour quality of 0.30, mass flux of 200 kg m^−2^ s^−1^, and saturation temperature of 35°C; (*d*) HTC response to (i) downward and (ii) upward step changes in inlet vapour quality; and (*e*) HTC response to (i) downward and (ii) upward step changes in inlet mass flux. Taken from Dirker *et al.* [37].

Having considered the impacts of varying the heat and mass fluxes on the HTC of flow boiling in pipes, Dirker *et al.* [37] proceeded to focus specifically on the investigation of vapour qualities in the range 15–40%, using refrigerant R-245fa as before. The apparatus, shown in figure 12, consists of a closed loop of the refrigerant R-245fa, circulated using a pump. The refrigerant flows from a liquid receiver, through a mass-flow meter, an electric preheater, electrically heated test section and a water-cooled condenser. The mass flow rate of the system was controlled via a bypass line and a needle valve. The facility used 12.7 mm outer-diameter smooth copper piping.

The role of time-varying climatic conditions, discussed previously in terms of their impact on the external heating (i.e. heat flux) conditions [35], has a knock-on effect on the ratio of liquid to vapour in pipes in solar fields. As the heating conditions in a CSP solar field change, the heat transfer to the fluid will be unsteady. The transient response was studied here by the authors via a series of imposed step changes. Results from the study by Dirker *et al*. [37] are shown in figure 12.

From this figure, we can see that the HTC varied non-uniformly when lowering or raising the vapour quality in such steps, with a higher-than-expected HTC when increasing the vapour quality and a lower-than-expected HTC when decreasing the vapour quality. This was attributed by Dirker *et al.* [37] to the flow pattern in the pipe taking the form of slug- and intermittent-flow regimes at lower vapour qualities and fluxes and an annular flow regime at higher vapour qualities [22]. It was suggested that future work should investigate higher vapour qualities than those considered here to consider annular flow regimes in more detail, and that a wider range of techniques for imagining fluids in pipe be applied to this experiment [23].

**Figure 13.**
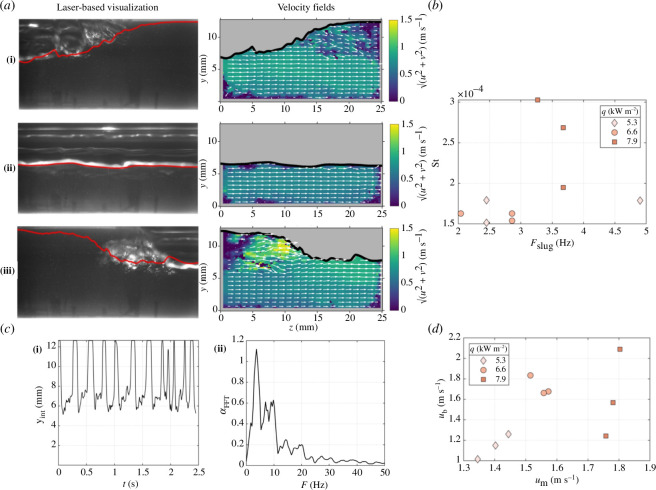
(*a*) High-speed images (left) and corresponding instantaneous velocity fields from PIV (right) in the pipe central plane at different streamwise positions along a vapour bubble: (i) tail, (ii) middle and (iii) front, for a flow with a mass flux of 460 kg m^−2^ s^−1^ and heat flux of 7.9 kW m^−2^, (*b*) dependence of the Stanton number (non-dimensional HTC) on the slug frequency for heat fluxes of 5.3, 6.6 and 7.9 kW m^−2^, (*c*) example time trace of the vertical position of the gas–liquid interface, and corresponding fast Fourier transform (FFT) and (*d*) dependence of the average velocity of vapour bubbles on the superficial mixture velocity for three flows with mass fluxes 320, 370 and 450 kg m^−2^ s^−1^ and corresponding heat fluxes 5.3, 6.6 and 7.9 kW m^−2^. Taken from Moran *et al.* [24].

In a final step, in terms of the development and application of advanced measurement techniques to these flows, Moran *et al.* [24] investigated flow boiling via the application of the laser-based techniques described in §4.2. At the investigated conditions, the flow was found to be in the ‘slug’ regime, whereby large bubbles dominate the flow. Selected results are shown in figure 13.

The authors considered a range of heat and mass fluxes and investigated their disturbances in the velocity fields in the flow. The frequency of slugs and their velocities were also measured. It was found that, at higher heat fluxes, which were set in this work to be in a range from 5.3 to 7.9 kW m^−2^, slugs in the flow were able to move faster than the bulk flow. It was suggested that these high-flux, high-speed slug regimes may give rise to an enhancement in the local turbulence levels in the flow compared with lower-heat-flux regimes.

For the case of flow boiling, further work by Moran *et al.* [24,25] involved the use of LIF (described above), along with PIV, to investigate the velocity within flows of R245fa simultaneously with pressure drop measurements from pressure gauges. In their experiments, the authors used R-245fa and a bespoke flow-boiling facility consisting of a flow circuit and an optical section for visualizations. Fluid was pumped from a liquid receiver and heated before entering the test section. A turbine flowmeter was used to measure the flow in the test section which was set/controlled via the pump speed, before the temperature and pressure were measured at the entrance to the heated section. A two-phase vapour–liquid flow is generated by boiling before the RIM visualization section enabled imaging of the flow.

**Figure 14.**
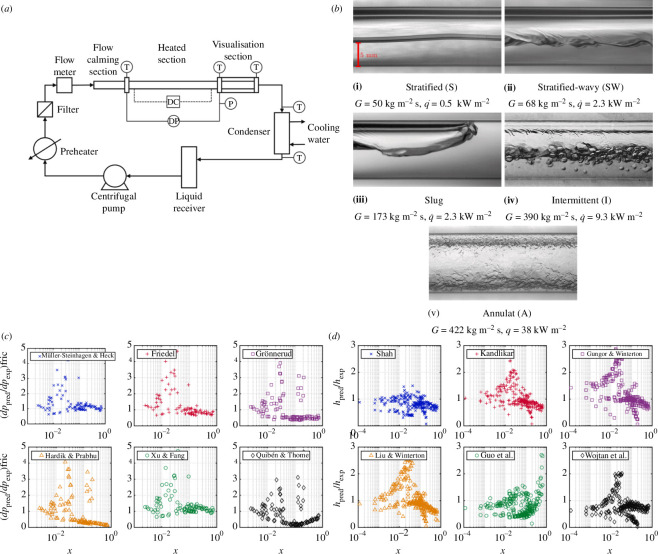
(*a*) Experimental flow-boiling apparatus, (*b*) flow patterns captured using high-speed imaging, (*c*) comparison of measured pressure drops against predictions from correlations and (*d*) comparison of measured HTCs against predictions as a function of vapour quality (*x*). Reproduced with permission from Moran *et al.* [25].

Of interest from the work performed here was an examination of how space- and time-resolved flow phenomena can impact the global, integral heat transfer and pressure drops in boiling flows. In this context, it was found that an enhanced understanding of the flow regimes could arise from this detailed information. Furthermore, the agreement between experimental results and theoretical predictions from well-known and commonly used correlations for the flow regimes, HTC and friction factors was explored in this work. Relevant comparisons are shown in figure 14, with the best agreement found between the experimental results and the predictions by Müller-Steinhagen & Heck [36] and Xu & Fang [38].

### 4.4. Computational studies

Concurrent with the experimental work covered above, the consortium undertook a range of computational studies of flow boiling and condensation with computational fluid dynamics (CFD) simulations. For example, after validating their code against the Barnea flow map [39] and finding good agreement, Juggurnath *et al*. [40] considered a range of pipe cross-sections—circular, square and rectangular pipes—aiming to categorize and analyse two-phase flow patterns, pressure drops and the void fraction within the flow. From these CFD simulations, a simplified model was developed for flows in circular, square and rectangular pipes.

Further research was performed by Juggurnath *et al.* [41] on the characteristics of condensing flows of the refrigerant R134a in horizontal pipes. Considering mass fluxes of 100, 200 and 400  kg m^−2^ s^−1^, a saturation temperature of 40°C, and mean vapour qualities of 0.25, 0.50 and 0.75, Juggurnath *et al.* [41] found that the HTC decreased around the pipe circumference from the top of the pipe to the bottom. The simulations performed by Juggurnath *et al.* [41] found an asymmetrical velocity profile in the condensing R134a flow which the authors attributed to the stratified condensate layer present at the bottom of the pipes. They also found that the mean flow velocity and HTC decreased along the condensation length.

#### 4.4.1. Integration with thermal energy storage and other technologies

DSG-CSP plants require thermal energy storage (TES) to enable the thermal energy generated to be used at hours other than when it is generated. The choice of TES technology, and its means of integration with CSP plants, can have significant impacts on the performance of the overall system as well as thermo-economic implications on the cost-effectiveness of installations. Alongside the computational work already discussed, the consortium investigated the integration of steam accumulation—a common technology choice for TES in DSG-CSP plants—with sensible-heat storage in concrete in order to provide higher-temperature steam at higher pressures [42].

**Figure 15.**
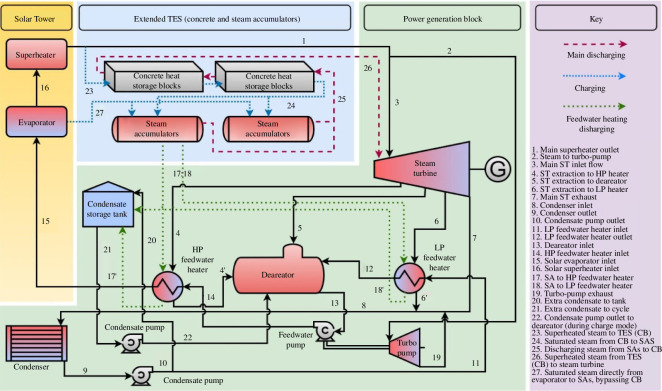
A schematic diagram of the Khi Solar One power plant considered by Al Kindi *et al.* [42] as part of their computational thermo-economic modelling of the plant with two different thermal-energy storage (TES) systems. Shown here is the configuration with the extended TES system proposed by Al Kindi *et al.* [42] consisting of the existing steam-accumulation system integrated with heat storage in concrete.

Al Kindi *et al.* [42] performed a thermo-economic analysis of this integration of steam-accumulation technology with concrete-based heat storage (a schematic of the plant design is shown in figure 15). The authors compared this integrated storage technique against a pure steam-accumulation TES system by modelling it as part of the Khi Solar One DSG-CSP plant in South Africa. The authors found that the integrated solution, when correctly optimized and configured, was able to use more of the thermal power delivered by the solar collector field, delivering 58% more electricity overall with a 13% increase in the thermal efficiency [42]. Economic analysis by Al Kindi *et al.* [42] found a 73% increase in the net present value of the case-study Khi Solar plant, assuming an average electricity price of USD280 MWh^–1^.

Having investigated the integration of DSG-CSP plants with TES, the consortium proceeded to consider the integration of CSP plants with other renewable-energy technologies. Owing to the diurnal variation of the output from solar-powered systems, Al Kindi *et al.* [43] considered the integration of DSG with nuclear (fission) power plants owing to their ability to provide renewable baseload electricity. The authors considered the integration of these two power-generation technologies with TES, taking NuScale (a modular nuclear reactor) and integrating it with CSP to consider the profitability of a hybrid DSG-CSP–nuclear plant and taking Oman as a case study for environmental data.

The authors used a molten-salt storage medium, with cold- and hot-tank temperatures of 260 and 565°C, respectively. Al Kindi *et al.* [43] found a levelized cost of electricity of USD78 MWh^–1^ compared with a wholesale price of USD43 MWh^–1^ in Oman (the case-study location) at the time of publication. They found, under full load and 75% partial load, that their hybrid nuclear–CSP system outperformed the standalone nuclear installation (NuScale) with efficiencies of 39% and 35% respectively, compared with the NuScale installation. They found solar heat-to-power efficiencies could reach as high as 56% for the combined system.

## 5. Conclusions

With the pressing need for sustainable, renewable energy worldwide, and in Africa and SSA in particular, the importance of developing research capacity and suitable technologies that promise to address this challenge cannot be overstated. Africa’s largely underutilized solar resource makes research into CSP highly relevant to the energy transition, specifically the United Nations’ SDG 7: affordable, reliable, sustainable and modern energy for all [8].

In response to this challenge, and motivated by the desire to promote sustainable development in SSA, the Royal Society, with funding from the UK’s DFID, supported a consortium of three universities across SSA (Lagos, Mauritius, Pretoria) and Imperial College London with the aim of developing new knowledge on CSP using DSG. The programme, titled ‘Harnessing unsteady phase-change heat exchange in high-performance CSP systems’, was one of ten such consortia supported by the £15 million Africa Capacity Building Initiative on water and sanitation, renewable energy, and soil-related science.

DSG-CSP plants produce steam directly in the solar field, thus bypassing the need for a secondary HTF (typically thermal oils or molten salts, depending on the temperature) and heat exchangers to generate steam for power generation. This allows higher steam temperatures, improved overall plant efficiencies, simplified designs, which are easier to maintain, reduced pumping-power requirements and reduced loss of HTF (through leaks) to the environment. DSG technology has attracted attention for these reasons; however, the operation of such plants under real, unsteady conditions that can arise from cloud coverage, ambient conditions and diurnal irradiance variations remains relatively unexplored, with a lack of knowledge on plant performance during transient operation.

The consortium investigated a range of thermal-fluid phenomena associated with boiling and condensation, such as those that would be used in key components of DSG-CSP plants in the field. An effort was made to quantify effects ranging from the appearance of different flow regimes in key components to the operation and performance capabilities of these components while subjected to the impact of variable conditions (e.g. heating or flow rates), as would be expected to arise from variable climatic conditions, in ways that had not been considered previously. Further research focused on identifying heat transfer enhancement opportunities, e.g. with surface engineering, as well as alternative avenues to mitigate the presence of unsteady operation at the plant level, e.g. by considering the role of energy storage.

In more detail, a new regime-classification scheme for two-phase, liquid–liquid flows was developed from an extensive database compiled from data in the existing literature, based on which the flow regimes expected to arise in relevant systems can be predicted *a priori* with improved reliability [22]. Furthermore, following a comprehensive review of appropriate (i.e. optically matched) solid materials and liquid substances for the design of dedicated experiments for the implementation of such measurements [23], the consortium was successful in the generation of a ‘first-of-a-kind’ phase and velocity-field information in boiling flows in horizontal pipes [24,25]. This effort necessitated, firstly, a systematic approach to develop and validate measurement techniques based on LIF and PIV/PTV methods, and a formal identification and quantification of the errors that arise in such measurements. For example, TIR when using PLIF techniques can lead to an overestimation of the film thickness in annular flows [26]. Alternatively, when using BBLIF, losses of signal sensitivity in thick film regions and the presence of steep or multiple interfaces also resulted in errors in the identification of the liquid–gas interface location.

The combined application of PLIF and BBLIF allowed a reduction in errors associated with the separate application of individual approaches, but also, based on these limitations, a new technique was proposed, referred to as S-PLIF, which overcame the above errors with uncertainties of 10% and 5% in the measurement of local/instantaneous and average film thickness in annular flows, respectively [27]. In a final step, Voulgaropoulos *et al.* [28] and Moran *et al.* [24,25] studied the pool boiling of water and the flow boiling (in the ‘slug-flow’ regime) of R-245fa in horizontal pipes via the combined application of the laser-based techniques developed in the project in order to obtain spatiotemporal phase distribution, velocity and temperature fields over a range of heat and mass fluxes.

Along with the development and application of the aforementioned techniques aimed at providing detailed, spatiotemporally resolved information in relevant flows, measurements of steady-state HTCs were performed in pool and film boiling aimed at exploring the heat-transfer enhancement potential of a range of modified surfaces. It was found that, in experiments of pool boiling over a range of reduced pressures (0.034−0.160), the commercial nanoFLUX surface outperformed copper-based surfaces in terms of the HTC, but that it also exhibited the highest variability over the range considered [29]. All surfaces exhibited power-law relationships between the HTC and pressure, with similar HTCs at the lowest pressure which then diverged at higher pressures. In a later study [33], the same surfaces were investigated for falling-film boiling for the refrigerants R-134a and R-245a. It was found that the nanoFLUX and oxidized-copped surfaces reached critical dryout at the lowest heat fluxes (~20 kW m^−2^), and that falling-film enhancement ratios typically reached values of 0.8−1.3. Overall, these results were important in understanding the benefits that surface modification (in particular, nanoengineering) can offer in the film boiling of refrigerants and pave the way for future studies on this topic using such approaches and for the deployment of such surfaces.

The programme had a particular interest in transient heat transfer as would be exhibited in real solar plants owing to varying solar irradiance or other environmental conditions. Therefore, research also focused on the response of HTCs for the refrigerant R-245fa to step, sinusoidal and triangular perturbations [35]. It was found that reductions in the HTC of 30, 8 and 8% occurred when the flow was subjected to step, sinusoidal and triangular perturbations, respectively, relative to steady-state expectations. Furthermore, Dirker *et al.* [37] found that the HTC deviated by between ~10 and ~30% from similar expectations during upward/downward steps.

Finally, Al Kindi *et al.* [42] performed a thermo-economic analysis of TES in the form of integrated (solid) concrete-based sensible-heat storage along with steam accumulation. They found that the TES-integrated plant, when correctly optimized and configured, was able to deliver 58% more electricity overall, with an increase of 13% in the thermal efficiency of the system and of 73% in its net present value [42]. In a later work, Al Kindi *et al.* [43] investigated the integration of DSG-CSP plants integrated with modular nuclear-fission reactors and found increases in the efficiency compared with a stand-alone nuclear facility of 39 and 35% under full and partial loads, respectively, suggesting significant synergies between these plants.

The findings have stimulated many further research questions, which will be important for the further exploration of the potential and challenges of this technology, but also in regard to using the capacity which was developed as part of the grant. Voulgaropoulos *et al.* [28] suggested that, in addition to the laser-based techniques used in their study, future work could include backlit shadowgraphy and an edge-determination algorithm to obtain the exact position of the liquid–vapour interface. They also identified a future research avenue, which could use the techniques they studied but with greater heating to examine faster-growing bubbles on more-heated nucleation sites. Bock *et al.* [29] recommended that future work consider the frequency and size of bubble formation to improve the understanding of the mechanisms for the enhancement of the HTC across the various surfaces. Al Kindi *et al.* [43] identified improvements to their steam-cycle modelling framework, as well as considering alternative hybrid configurations, as avenues for future work based on their modelling framework. Future work that used the data gathered to inform future studies was suggested by Van den Bergh *et al.* [35].

Through the transfer of knowledge and skills, the partner institutions in the consortium were able to support the establishment of a research capacity base alongside the training of a cohort of young, talented African researchers (through PhD scholarships) and technicians. Such a base has the potential to give rise to future opportunities, such as novel future research and new business opportunities, leading to a positive impact on local and national economies and societies as well as co-benefits to people living in the region.

By the end of the project, the grant successfully reported new research findings relating to DSG-CSP technology, and established self-sustaining research networks across the consortium institutions in these countries that will be capable of undertaking high-quality, international research in similar future projects with a global reach.

## Data Availability

Data supporting this publication can be obtained on request from cep-lab@imperial.ac.uk. For the purpose of Open Access, the authors have applied a CC BY public copyright licence to any Author Accepted Manuscript version arising from this submission.
